# The Effect of Straw, Rope, and Bite-Rite Treatment in Weaner Pens with a Tail Biting Outbreak

**DOI:** 10.3390/ani9060365

**Published:** 2019-06-17

**Authors:** Helle Pelant Lahrmann, Julie Fabricius Faustrup, Christian Fink Hansen, Rick B. D’Eath, Jens Peter Nielsen, Björn Forkman

**Affiliations:** 1 SEGES, Danish Pig Research Centre, Agro Food Park 15, 8200 Aarhus, Denmark; CFHA@SEGES.dk; 2 Department of Veterinary and Animal Sciences, University of Copenhagen, Grønnegårdsvej 8, 1870 Frederiksberg, Denmark; jffaustrup@gmail.com (J.F.F.); jpni@sund.ku.dk (J.P.N.); bjf@sund.ku.dk (B.F.); 3 SRUC, West Mains Road, Edinburgh EH9 3JG, UK; rick.death@sruc.ac.uk

**Keywords:** pigs, swine, weaners, behaviour, tail injury, tail biting outbreak, enrichment material, straw, rope, Bite-Rite

## Abstract

**Simple Summary:**

Young pigs can bite each other’s tails, which is a welfare problem. It begins suddenly and spreads like an “outbreak”. Pig farmers use various methods to prevent tail biting, but if prevention fails, a cure is needed, and there has been little scientific research into how best to stop an outbreak. In a study with 65 groups of young pigs, we tested three methods of stopping tail biting outbreaks which could be practical to use on commercial farms: (1) straw (small amount on the floor), (2) rope, and (3) Bite-Rite (a hanging plastic device with chewable rods). All provided some distraction, but straw stopped an increase in tail injuries more often (75%) than the Bite-Rite (35%), with rope intermediate (65%). Watching the pigs’ behaviour showed that they preferred to interact with rope than the Bite-Rite. We also saw that interacting with other pigs’ tails increased after a week with the Bite-Rite but not with rope or straw. Overall straw worked best, but future studies may find even more effective ways to stop tail biting outbreaks, once they begin.

**Abstract:**

Tail biting in pigs is an injurious behaviour that spreads rapidly in a group. We investigated three different treatments to stop ongoing tail biting outbreaks in 65 pens of 6–30 kg undocked pigs (30 pigs per pen; SD = 2): (1) straw (7 g/pig/day on the floor), (2) rope, and (3) Bite-Rite (a hanging plastic device with chewable rods). Pigs were tail scored three times weekly, until an outbreak occurred (four pigs with a tail wound; day 0) and subsequently once weekly. After an outbreak had occurred, a subsequent escalation in tail damage was defined if four pigs with a fresh tail wound were identified or if a biter had to be removed. Straw prevented an escalation better (75%) than Bite-Rite (35%; *p* < 0.05), and rope was intermediate (65%). Upon introduction of treatments (day 0), pigs interacted less with tails than before (day −1; *p* < 0.05). Behavioural observations showed that pigs engaged more with rope than Bite-Rite (*p* < 0.05). Bite-Rite pigs (but not straw or rope) increased their interaction with tails between day 0 and day 7 (*p* < 0.05). Straw was the most effective treatment. However, further investigations may identify materials or allocation strategies which are more effective still.

## 1. Introduction

Tail biting in pigs is an abnormal behaviour and has been reported both in conventional and in free-range/organic productions [1,2]. Tail biting by single pigs may, if not identified in the early stages, develop into a tail biting outbreak [3]. European Food Safety Authority (EFSA) [4] defined a tail biting outbreak as occurring when the tail biting intensifies, leading to several tail damaged pigs and the tail biting will continue, if an intervention is not conducted. If the tail biting continues within a group of pigs, the biting behaviour may lead to severe injuries with tail loss and infections followed by carcass condemnation at the abattoir [5].

It is well established that giving pigs access to enrichment reduces tail damage [6,7,8,9]. However, although there has been considerable research into risk factors and preventive treatments, only one experimental study, Zonderland et al. [8], has investigated the curative effect of interventions in pens with a tail biting outbreak. Systematic studies evaluating the effect of different curative treatments in pens with a tail biting outbreak are therefore needed [3,9]. Curative treatments in this context refer to interventions aiming at stopping or reducing tail biting behaviour and tail damage in pens after a tail biting outbreak has already started. The most frequently studied preventive enrichment material is straw on the floor in various amounts (reviewed by D’Eath et al. [9], Brunberg et al. [10]). To avoid the problems of loose straw in fully or part-slatted systems as either a preventive or curative treatment, hanging materials could be used. However, the effect of hanging materials on tail damage during an outbreak has not been investigated in previous studies (reviewed by D’Eath et al. [9]). D’Eath et al. [9] discussed that in order to avoid waste, as well as problems with straw blocking the slats and slurry system, litter material such as straw is only practicable in pens with a solid or part-solid floor and in small amounts. Other solutions are therefore needed for production systems without a solid floor to stop tail biting, once an outbreak has begun.

Scientific knowledge of the efficacy of different intervention strategies as curative measures for tail damage during a tail biting outbreak is crucial to reduce the negative welfare impact of the outbreak and to support evidence-based advice for farmers. The aim of the present study was therefore to examine the effect of either a small amount of straw on the floor, rope, or Bite-Rite (a hanging plastic device with chewable rods) on tail damage and behaviour in pens with a tail biting outbreak. These enrichment types were chosen due to their possible practical implementation, if the material successfully ceased tail damage. As previous studies reported, less use of enrichment in pens with Bite-Rite compared to straw [11] and because rope is more destructible, it was hypothesised that straw on the floor and rope would reduce tail damage more efficiently than Bite-Rite.

## 2. Materials and Methods

This experiment was a continuation of the work presented in Lahrmann et al. [12] using the same study subjects. However, while Lahrmann et al. [12] dealt with identifying behavioural changes before tail biting outbreaks, the current study focused on the effect of interventions in pens with tail biting outbreaks. The study was carried out at a commercial piggery from November 2015 to February 2016.

### 2.1. Ethical Consideration

This experiment was conducted in accordance with the guidelines of the Danish Ministry of Justice, Act No. 382 (June 10, 1987) and Acts 333 (May 19, 1990), 726 (September 9, 1993) and 1016 (December 12, 2001) with respect to animal experimentation and care of animals under study. Experiments done in accordance with the Danish legislation on animal husbandry do not require further ethical permissions.

### 2.2. Experimental Design

The study was designed to compare the effect of three different curative treatments on tail damage and behaviour in pens with a tail biting outbreak. On the day of the tail biting outbreak (defined as day 0; see Section 2.4 for outbreak definition), one of three treatments were randomly allocated to the pen: straw, rope, or Bite-Rite. To follow the development in tail injuries, tails were scored once weekly after an outbreak was noted and the behaviour of the pigs was recorded on day −1, 0, 2, and 7.

### 2.3. Animals and Housing

This study included 1987 undocked DanBred crossbred ((Landrace × Large White) × Duroc) nursery pigs in 65 pens as they grew between approximately 6–30 kg. Pigs originated from four different farrowing batches with 458–525 pigs per batch. Pigs were born in a farrowing system with loose sows (for pen design details, see Pedersen et al. [13]). Iron injections (Uniferon, Pharmacosmos, Holbæk, Denmark), grinding of the needle teeth (Tandsliber proff, Hatting, Horsens, Denmark) and surgical castration of male piglets with a scalpel were carried out on day 3 or day 4 after birth. Male piglets were given analgesic just before castration (Melovem^®^ 5 mg/mL, Raamsdonksveer, The Netherlands). Throughout the lactation period, piglets had access to the straw that the sow pulled from a straw rack. Approximately two weeks after farrowing, piglets were offered solid feed on the floor in the creep area. Two days before weaning, the pigs were ear tagged and their sex were noted. The lactation period was 27.8 days (SD = 2.9) and pigs weighed 5.8 kg (SD = 1.5) at weaning. After weaning, pigs were transported to a nursery facility close to the sow facility (1.5 km).

At the nursery facility, pigs were sorted by size within a batch and randomly allocated to nursery pens with 30 pigs/pen (SD = 2; 0.35 m^2^/pig). Sex distribution was on average 49.2% (range = 32.2% to 77.4%) gilts per pen. The four experimental rooms consisted of 26 or 30 pens, and between 18–20 pens per room were included in the experiment. Pens measured 4.85 × 2.18 m (length × width) with 7.1 m^2^ solid floor and 3.5 m^2^ cast iron slatted floor. A 2.16 m^2^ adjustable covering was placed above the solid floor in the lying area. Adjacent pens shared a dry feed dispenser with two nipple drinkers, one placed on each side of the feed dispenser (MaxiMat Weaner 7–60 kg, Skiold A/S, Sæby, Denmark). Additionally, a separate water supply (drinking bowl) was placed next to the feed dispenser towards the slatted floor. Each pen was equipped with two wooden blocks hanging from a chain just above the floor without touching the floor to ensure permanent access to enrichment according to legislation [14]. Pens were provided daily with approximately 350 g of finely chopped straw (Easy Strø, Easy Agri Care, Nykøbing Mors, Denmark) on the solid floor. Artificial lighting was on from 06:00 h–22:00 h.

The rooms were ventilated by a negative pressure air flow through wall air inlets on one side of the building (SKOV A/S, Glyngøre, Denmark). The room temperature at weaning was 24 °C and was gradually lowered to 19 °C on day 42. Thermostatically controlled floor heating pipes were placed inside the floor in the lying area, giving a floor temperature of 30 °C at the start of the study. The floor heating was turned off on day 14. 

Pigs were fed three different home-mixed compound diets (ad libitum access) from 6–30 kg. The diets were formulated to fulfil the nutritional requirements of pigs of this age and genotype. Phase 1 diet allocated from approximately 6–10 kg (17.4% crude protein) consisted of 64.0% wheat, 22.0% premix of minerals and vitamins (HeavyPig 3 20%, Vilomix, Mørke, Denmark), 10.5% fish meal, and 3.5% soy oil. Phase 2 diet allocated from approximately 10–15 kg (18.1% crude protein) consisted of 44.4% wheat, 25.0% barley, 15.0% toasted soy bean, 8.2% premix of mineral and vitamins (MIN 27600, Vilomix, Mørke, Denmark), 5.0% fish meal, and 2.4% soy oil. Phase 3 diet allocated from approximately 15–30 kg (18.4% crude protein) consisted of 48.8% wheat, 25.5% toasted soy bean, 20.0% barley, 4.2% premix of mineral and vitamins (MIN 27603, Vilomix, Mørke, Denmark), and 1.5% soy oil. The transition between feed compounds depended on the average body weight in the pen at weaning and was gradually conducted over a 7- or 14-day period. Pigs were housed at the nursery location for 6.5 weeks before being moved to the finisher facility, at which point they left the study.

During the stock person’s daily inspection, pigs with clinical signs of disease were treated with antibiotics when needed according to the herd veterinarian’s recommendations. Unthrifty animals and pigs with severe tail lesions (more than half the tail missing or swelling as a sign of infection) were moved to hospital pens.

### 2.4. Tail Scoring and Tail Biting Outbreak (Day 0)

From weaning until a tail biting outbreak, tails were scored three times weekly (Monday, Wednesday, and Friday) to determine the day of the outbreak. Tail damage severity and tail posture were recorded in the same way as in Lahrmann et al. [12] by researchers. During tail scoring, ear tag number, tail damage severity, wound freshness, tail length, and tail swelling on injured tails were recorded on each individual according to the criteria listed in Table 1.

A “tail biting outbreak” occurred when at least four pigs in a pen (approximately 13% of the pigs) had a tail wound (see definition of a wound in Table 1) irrespective of “damage freshness”, “tail length”, and “swelling”. A wound was more severe than “minor scratches”. The day of the tail biting outbreak was set as day 0. The stock person did not observe any tail biting outbreaks during the daily inspection of pigs between recording days.

In pens with a tail biting outbreak, tails were scored once weekly on day 7, day 14, day 21 and so on after the outbreak. Tail scoring continued, until an escalation in tail damaged pigs was observed (see definition in the curative treatment paragraph below).

### 2.5. Treatments

One of three curative treatments selected in a predetermined random order, was allocated to the pen on the day of the tail biting outbreak (day 0): straw (23 pens), rope (22 pens) or a commercially available hanging plastic enrichment with four chewable rods (Bite-Rite, Ikadan Systems A/S, Ikast, Denmark, 20 pens). A curative treatment was provided in 65 pens and it was maintained until the pigs were moved to the finisher facility (study end).

In pens with straw, approximately 200 g (7 g/pig/day) of chopped wheat straw (chopped during harvest in a combine harvester) were provided on the solid floor once a day. This was in addition to the 350 g of finely chopped straw, which all pens received daily throughout the study period.

In pens with rope, a coil of sisal rope (20 mm in diameter) was placed above the pen. The rope was pulled from the coil leaving roughly 30 cm of rope on the solid floor, and the top end at the coil was secured, ensuring that no more rope could be pulled out by the pigs. The rope was provided in the middle of the pen, approximately one meter from the slatted floor. A knot was tied about 20 cm from the rope end to reduce consumption. If the rope end was consumed, the knot was loosened, and new rope was provided the following day in the same way as described above.

In pens with a Bite-Rite, the device was suspended in the middle of the pen at the same location as rope. The plastic rods were located at a height at which pigs could easily reach and chew on them—standing or sitting. As the pigs grew, the Bite-Rite was gradually raised.

### 2.6. Defining Escalation in Tail Damage and Removing Biters and Victims

Two criteria were used to determine if the curative treatment failed to stop the tail biting behaviour; an escalation in fresh wounds (direct measure) and removing a biter (indirect measure). Fresh wounds were tail damage more severe than “minor scratches” and a fresh wound was either weeping or bleeding according to “damage freshness” (see description in Table 1). This definition of an escalation was chosen, as we wanted a definition where it was clear that the tail biting was ongoing. Removing the biting pig was used as a criterion as this indirect measure also reflected whether the curative treatment served the primary purpose; to stop the tail biting behaviour. When the term “an escalation in tail damage” is applied in this paper, it refers to the sum of pens in which a biter was removed, and pens observed with four fresh wounds or more, either of which imply a failure of the curative treatment to stop the tail biting behaviour. Pens observed with four fresh wounds or pens from which a biter were removed were excluded from the study at this point. In these pens, extra steps were taken to stop the biting behaviour to ensure the welfare of the remaining pigs, such as providing other or more enrichment material.

The four fresh tail wounds and biters could either be observed during weekly tail inspection days (see Section 2.5) or during the daily health inspection. Biters were pigs observed repeatedly chewing/biting the tail so hard that the receiver screamed and reacted by suddenly moving away or turning against the biting pig. Pigs performing this kind of behaviour were removed from the pen.

Tail biting victims with severe tail lesions defined as more than half the tail missing or swelling as a sign of infection were moved to a hospital pen for appropriate veterinary treatment and monitoring.

### 2.7. Behavioural Recordings on Day −1, 0, 2, and 7

An overhead surveillance video camera (Dahua 2MP HD IR Dome, Dahua, Haarlemmermeer, The Netherlands) was installed above each pen timed to record from 07:00 h to 21:00 h. The time was chosen based on a previous study showing that pigs are most active in the day time [15]. Video data were collected from when the pigs entered the pen and ended when data collection stopped due an escalation in tail injuries or when pigs were moved to the finisher unit.

Behavioural observations were made, using video recordings on day −1 (the day before a tail biting outbreak and introduction of the enrichment treatment), day 0, day 2, and day 7. Not all 65 pens could be included in the behavioural study, either due to poor video quality or due to an escalation in tail biting during the sampling period. As such, behavioural observations were conducted using video from 33 pens (straw *n* = 11, rope *n* = 14, and Bite-Rite *n* = 8).

The behavioural observations were divided in two parts. In the first part, the number of pigs engaged in object interaction or other behaviours (as listed in Table 2) performed by standing, sitting, or lying pigs were recorded in the pen using scan sampling once every 30 min between 07:00 h and 21:00 h. In the second part using continuous sampling, the frequency of tail directed behaviour (TDB) was recorded at the pen level as listed in Table 3 during a 10-min period every 2nd hour between 07:00 h and 21:00 h, starting at 07:00 h. Tail interest (TI) was recorded if it lasted more than one second, and if the pig repeated the behaviour after a pause of 2–3 s, it was recorded as a new incidence. Additionally, if the pig shifted between two types of tail directed behaviour towards the same recipient, both behaviours were recorded, for example: tail interest, to tail-in-mouth, further to two-stage tail biting, and back to tail interest after the recipient pig reacted to tail biting; a total of four incidences were recorded.

### 2.8. Statistical Analysis

Statistical analyses were performed in SAS Enterprise Guide 7.1 (SAS Institute Inc., Cary, NC, USA) with a significance level of *p* < 0.05.

#### 2.8.1. Escalation in Tail Injuries 

An intervention was conducted in 65 pens, but in four pens, the tail biting outbreak occurred within the last week of the experiment (straw *n* = 1, rope *n* = 2 and Bite-Rite *n* = 1). These four pens were excluded from the analyses because the effect of the curative treatment could only be tested for less than one week.

The difference in the number of tail damaged pigs between treatments on day 0 and the effect of the number of tail damaged pigs on day 0 on the risk of an escalation in tail damage was analysed using the general linear mixed procedure (GLIMMIX). The treatment and days after weaning until outbreak were fitted as fixed effects and batch as random effect.

The GLIMMIX procedure was also used to analyse the effect of treatment (straw *n* = 22, rope *n* = 20 or Bite-Rite *n* = 19) at pen level on a potential escalation (binary outcome) in tail damage (removing biter or at least four fresh wounds). In this analysis, treatment and days until outbreak were fitted as fixed effect and batch as random effect.

#### 2.8.2. Tail Damage Severity

Tail injuries were grouped according to severity and tail length, but irrespective of damage freshness (0 = no tail damage, 1 = tail damage and full tail length (mild), 2 = tail damage and tail loss or swollen tail (moderate)) before statistical analysis. The difference in the number of tail damaged pigs between treatments on day 7 and day 14 in pens (*n* = 27) without an escalation in tail injuries was analysed using the GLIMMIX procedure. The treatment, the days after weaning until outbreak, and tail damaged pigs on day 0 (and day 7 in the model analysing differences on day 14) were fitted as systematic effects and batch as random effect in the model.

The percentage of pigs with tail damage (category 1 or 2) on day 0, day 7, and day 14 in pens without a subsequent escalation in tail injuries irrespective of treatment and that had been at the facility for at least 14 days after treatment start were analysed (*n* = 27) using the mixed linear procedure (MIXED) with days after weaning until outbreak and day after intervention (day 0, 7, or 14) as fixed effect. Batch and pen were included as random effects.

#### 2.8.3. Behavioural Observations 

Behavioural recordings were conducted in 33 pens. Some video sequences were disturbed or not available. Missing sequences accounted for 1.8% of the total planned scans and 2.6% for continuous sampling.

The percentage of active pigs was calculated as the proportion of the total number of pigs in the pen. The other behaviours recorded using scan sampling were calculated as the proportion of active pigs (sum of walking, standing, sitting active, and lying active). The proportion of pigs engaged in hanging object interaction was analysed for the Bite-Rite and rope treatment. In pens with the straw treatment, it was not possible to distinguish between explorative behaviour directed against the straw and floor. Therefore, in order to compare the three treatments, exploratory behaviour towards the floor, substrate and hanging object were summed. Behavioural recordings collected by scan sampling were analysed using the MIXED procedure. No interaction between treatment and day was present.

Tail directed behaviour (TI, tail-in-mouth (TIM), and tail biting (TB)) were analysed as mean frequencies per day within the observation period (80 min per day). Due to very low frequencies, sudden-forceful-biting, and two-stage-biting were grouped together. Tail directed behaviour (continuous sampling) was analysed using the GLIMMIX procedure with day and treatment (straw *n* = 11, rope *n* = 14, Bite-Rite *n* = 8) as fixed effects and pen × treatment and batch as random effects. Tail directed behaviour estimates were back transformed using the i-link function. The analysis demonstrated an interaction between treatment and day.

Results from analysis in the GLIMMIX and MIXED procedure are presented as least square means (LSmean) and standard error (SE).

## 3. Results

**Figure 1:**
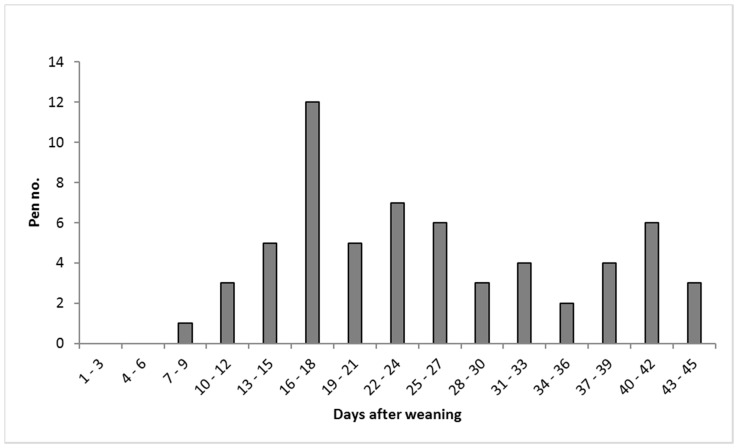
Incidence of first tail biting outbreak at pen level during the study period (*n* = 61).

On average, a tail biting outbreak developed 25 days (SD = 10.2; range = 9–45 days; median *=* 23 days) after weaning (Figure 1), and 6.8 pigs/pen (SD = 3.4; range = 4–21 pigs) had tail damage at that timepoint (day 0). An escalation in tail damage (four fresh tail wounds or removing a biter; *n* = 25) occurred on average 14 days (SD = 9.2; range = 2–34 days) after the outbreak. See Table 4 with descriptive results presented by treatment. In four pens of the 25 pens with an escalation in tail damage, removing the biter was the reason, whereas in 21 pens, the incidence of four fresh wounds was the cause (Table 4).

### 3.1. Effect of Curative Treatments on Escalation in Tail Damage

In total, 61 pens were included in the statistical analysis (straw *n* = 22; rope *n* = 20; Bite-Rite *n* = 19). On day 0, when one of the three curative treatments were provided, the number of tail damaged pigs at pen level did not differ between treatments (*F*_2,55_ = 0.19, *p* = 0.83) and the number of tail damaged pigs on day 0 did not affect the risk of a subsequent escalation in tail damage (*F*_1,55_ = 0.10, *p* = 0.76). 

**Figure 2:**
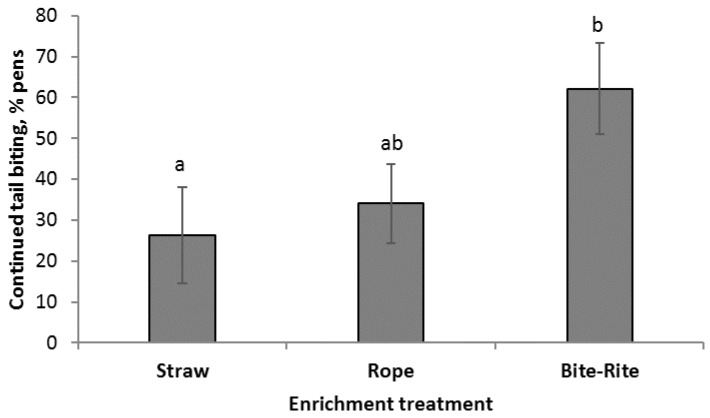
Percentage of pens with an escalation in tail damage after the initial outbreak presented as least squares mean (LSmean) (±standard error (SE)) according to treatment. Different superscript (a, b) indicates a significant difference of *p* < 0.05 between treatments.

An escalation in tail damage (sum of removed biting pig and pens with four fresh wounds) occurred in more pens with Bite-Rite than in pens with straw (Figure 2). There were no significant differences between rope and either Bite-Rite or straw.

In total, fewer pigs received tail damage in straw pens (16.7%) compared to Bite-Rite (25.6%; *t*_(1811)_ = 3.81, *p* < 0.001) and rope (22.8%; *t*_(1811)_ = 2.69, *p* < 0.01). Whereas no difference in tail damaged pigs was observed between rope and Bite-Rite pens (*t*_(1811)_ = 1.12, *p* = 0.26).

### 3.2. Effect of Curative Treatments on Behaviour

There was no difference in activity between treatments, but pigs were more active on the day of the outbreak (day 0) than on the other days (Table 5). More behaviour was directed towards the rope than the Bite-Rite, and more behaviour was directed against the objects on day 0 than on day 2 and day 7 (Table 5). More exploratory behaviour was observed in pens with rope than in pens with Bite-Rite and straw, and more exploratory behaviour was observed on day 0 than on day −1, 2, and 7 (Table 5). Furthermore, the level of pen mate directed behaviour (excluding tail-directed behaviour) was lower on day 0 and 2 than on day −1. No difference in pen mate directed behaviour was observed between treatments.

**Figure 3:**
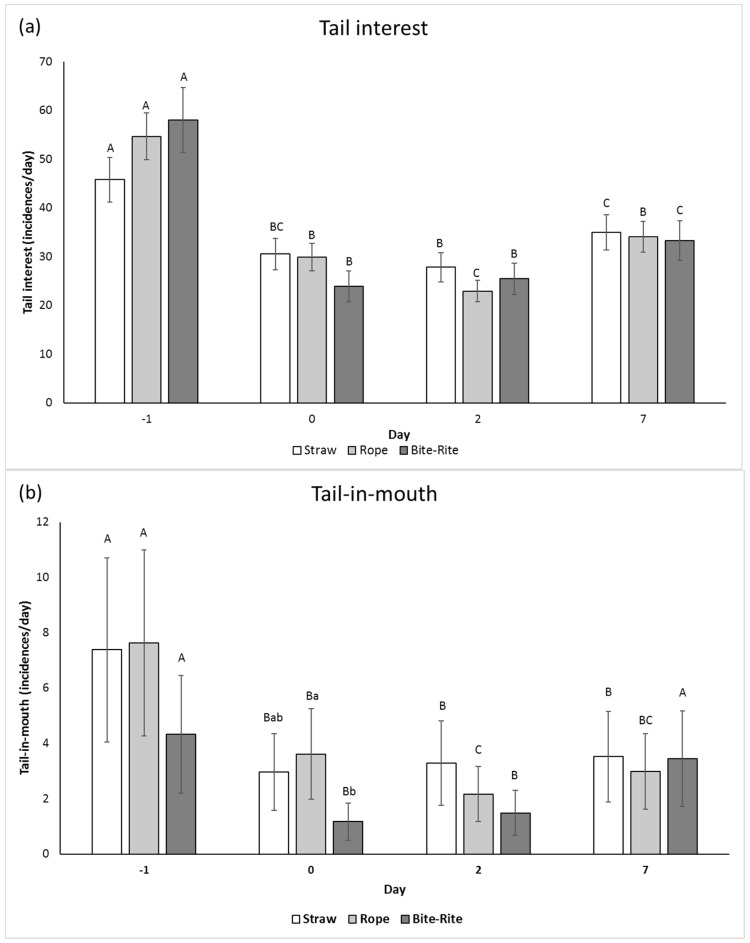

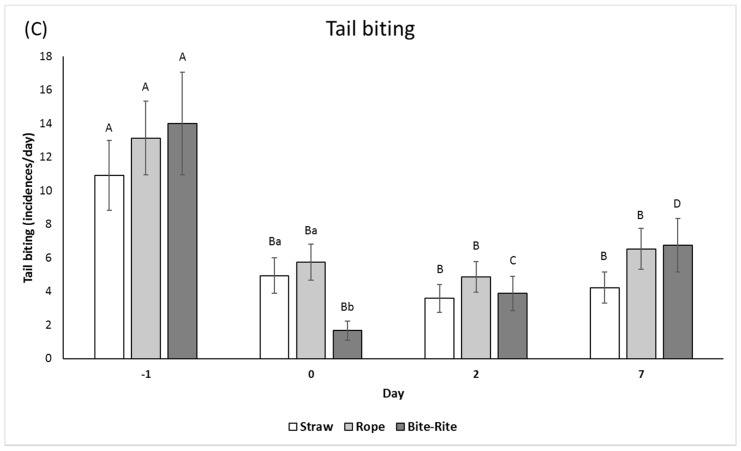
Tail interest (**a**), tail-in-mouth (**b**), and tail biting (**c**) presented as frequency per day (LSmean ± SE) for each enrichment treatment (straw, rope and Bite-Rite) on day −1, 0, 2, and 7 (80 min observation period per day). Different lowercase letters indicate a significant difference of *p* < 0.05 between treatments within days. Different uppercase letters indicate a significant difference of *p* < 0.05 between days within treatments.

Figure 3 shows the effect of enrichment treatment on tail interest (TI), tail-in-mouth (TIM), and tail biting (TB) on day −1, 0, 2, and 7. Irrespective of enrichment treatment TI, TIM, and TB were lower on day 0 than on day −1. Furthermore, except for TIM on day 7 in Bite-Rite pens, the frequency of TI, TIM, and TB was lower on day 2 and 7 than on day −1. In pens with Bite-Rite, the frequency of TI, TIM, and TB was higher on day 7 than on day 0 and 2. There was no difference in TIM and TB in rope and straw pens on day 7, day 0, or day 2, but TI was observed more often in rope and straw pens on day 7 than on day 2, but not on day 0.

TB differed between treatments on day 0 with a lower prevalence in pens with Bite-Rite than in pens with rope and straw, and TIM was lower in pens with Bite-Rite than in pens with rope on day 0.

### 3.3. Tail Damaged Pigs on Day 0, 7, and 14 after Curative Treatment in Pens without an Escalation

**Figure 4:**
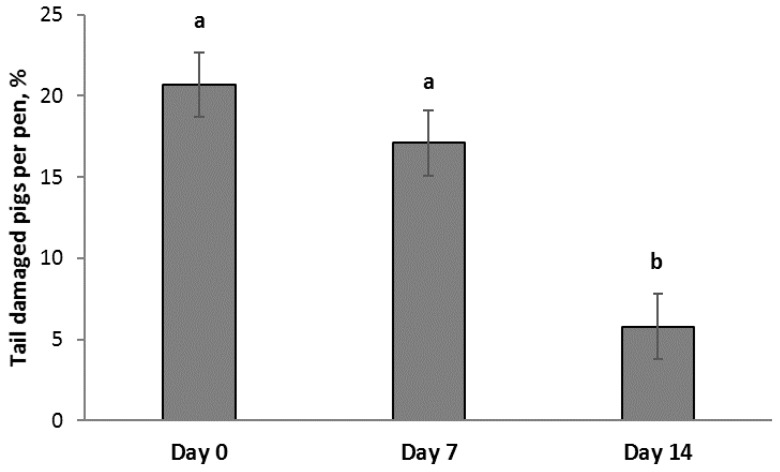
Percentage of tail damaged pigs per pen on day 0, day 7, and day 14 (LSmean ± SE) after the intervention in pens without an escalation in tail damage (n = 27). Different superscripts (a, b) indicate significant difference of *p* < 0.001.

No difference in the number of tail damaged pigs was observed between treatments on day 7 (*F*_2,21_ = 0.08, *p* = 0.92) or on day 14 (*F*_2,20_ = 1.0, *p* = 0.37) after the first outbreak in pens without an escalation in tail damage. Combining data from the three treatments with no subsequent escalation in tail damage (n = 27 (straw *n* = 11, rope *n* = 11 and Bite-Rite *n* = 5)), 20.7% of the pigs per pen (6.4 pigs per pen; SD = 3.5) had tail damage on day 0. In these pens, fewer pigs had tail damage on day 14 (5.8%; 1.7 pigs per pen; SD = 2.1) than on day 0 and day 7 (17.1%; 5.0 pigs per pen; SD = 3.5), but there was no difference between day 0 and day 7 (Figure 4).

## 4. Discussion

This is, to our knowledge, the first study to compare the effect of straw, rope, and Bite-Rite as curative treatments during an ongoing tail biting outbreak (see also Lahrmann et al [20]). An escalation in tail damage was observed in 26% of the straw pens, 34% of the rope pens, and 62% of the Bite-Rite pens. Providing a small amount of straw on the floor during an ongoing tail biting outbreak reduced the risk of a further escalation in tail damage more efficiently than providing a Bite-Rite. Numerically, rope fell between the other two treatments, but no significant difference was demonstrated between pens provided with rope and pens provided with straw or Bite-Rite.

On average, a tail biting outbreak occurred 23 days after weaning (range = 5–45 days), which is in line with Zonderland et al. [8] reporting tail biting outbreaks in 50% of the pens with undocked tails (median) 24 days after weaning (range = 8–31 days). In agreement, Veit et al. [21] reported that tail damage began to occur two to three weeks after weaning in pens with undocked tails, while D’Eath et al. [22] reported a mean outbreak day of 28 days post-weaning (range = 16–41 days).

In pens with rope, pigs interacted significantly more with the object than in pens with Bite-Rite. These findings are in line with a review by Studnitz et al. [23] concluding that pigs’ preference towards an object will increase with its destructibility and complexity. As the rope end laid on the floor, the material might have been available to more pigs and the material was more destructible than the hanging Bite-Rite. Both in rope and Bite-Rite pens, a decline in object interaction was observed on day 2 after the intervention. This indicates that the effect of novelty decreased within a few days. Other studies including a Bite-Rite demonstrated similar results with a higher level of enrichment manipulation in pens with straw than in pens with Bite-Rite [24,25]. In pens with rope, pigs performed more exploratory behaviour than in straw and Bite-Rite pens. The provided straw gradually disappeared over time and was therefore not permanently present, which could explain the higher level of exploratory behaviour in pens with permanent access to rope on the floor.

Irrespective of treatment tail interest, tail-in-mouth and tail biting declined from day −1 to day 0 and day 2. Bite-Rite caused the greatest reduction in tail biting from day −1 to day 0, compared to the other two treatments. However, each of the tail-directed behaviours increased during the following seven days in Bite-Rite pens, while the frequency remained more constant in pens with rope or straw. It may indicate that the attractiveness of Bite-Rite was higher at first, but faded quicker than the other two materials, which gave rise to more tail-directed behaviour on day 7. This is in line with a study by Van de Weerd et al. [25] reporting more tail biting in pens with a Bite-Rite than in pens with straw bedding.

In pens without a further escalation in tail damage, fewer pigs had tail damage on day 14 than on day 0 and day 7. This suggests that it took more than seven days for a tail wound to heal with the allocated enrichment materials. These results are supported by Lahrmann et al. [26] reporting that 89% of the tail wounds healed within 14 days. However, as discussed by Lahrmann et al. [26], the duration of the wound healing is undoubtedly affected by the severity of the wound.

In the present study, even if the most efficient curative treatment (7 g/pig/day of chopped straw on the pen floor) was applied, the tail damage escalated in approximately one in four pens. A different intervention strategy or other kinds of interventions are likely needed to stop the biting behaviour even more efficiently during a tail biting outbreak. These interventions could include removing tail damaged pigs, providing the materials used in the present study in combination, providing materials in a greater quantity, providing other types of enrichment or perhaps shifting between enrichment materials during the post-outbreak phase. In a study by Zonderland et al. [8], an allocation of straw twice daily (20 g/pig/day) or removing the biting pig as curative treatments were equally likely to stop the tail biting. In the Zonderland study, the treatment effect was measured as the prevalence of pigs with a fresh wound in the following ten days after the tail biting outbreak. However, ten days after the intervention, 11% of the pigs still had a fresh tail wound, compared with 25% on the day of the outbreak, suggesting that even these interventions did not stop the biting behaviour completely.

The curative treatments applied in the present study were chosen due to their feasibility in current production systems. In the present study, straw as curative treatment was given additionally to very finely chopped straw provided on a daily basis. It could be that providing a larger amount of straw and thereby ensuring access for a longer period of the day would have reduced the escalation in tail biting more efficiently [27]. Hence, increasing amounts of straw, increases the time pigs interact with the material [28,29]. There are, however, practical problems with larger amounts of straw, as it increases the risk of the material accumulating in the slurry canals or blocking up the slurry pipes [9]. To ensure access to straw for a longer period during the day, Oxholm et al. [28] demonstrated that more frequent allocation (four times daily vs. once a day) of the same total amount of straw ensured more straw left in the pen the following day. Another approach could be to give the straw in a rack, which would probably also increase accessibility. However, straw in a rack might not be as effective as straw on the floor, if pigs have difficulty pulling the straw from the rack and on to the floor [8].

Another possibility could be to allocate a material that pigs find more attractive than straw. In a review by Studnitz et al. [23], more complex materials (mixtures) were ranked higher than straw, when evaluated according to attractiveness. These materials might stop tail biting more efficiently, because pigs find them more attractive even in smaller amounts. However, tail biting studies like the current one are required to establish the materials’ effect during a tail biting outbreak.

In pens provided with rope, a knot was tied approximately 20 cm from the rope end. The rope treatment might have prevented an escalation in tail damage to a greater extent without the knot. When rope beneath the knot was consumed, the rope was no longer destructible and chewable. This may have caused less interaction with the material, until new rope was released the following day. It may also be that several pieces of rope are needed to stop the biting completely. This would give more pigs access to the material simultaneously and may avoid pigs experiencing frustration due to lack of access to the material, as discussed by Docking et al. [30]. However, in a minor study by Scott et al. [31] (eight pens per treatment), access to four toys vs. one toy made of alkathane piping did not increase the proportion of observations at which pigs interacted with the material.

Novelty, besides destructibility and manipulability, is an important feature to keep pigs interested [23]. Therefore, a shift between different types of hanging materials could have been more effective in reducing tail biting. Perre et al. [32] reported in a minor study (six pens per treatment) that shifting between hanging enrichment objects reduced tail damage and biting behaviour compared to only providing a chain.

Tail biting occurs sporadically [9], which makes the planning and control of such a study difficult. In addition, while studies have to be scientifically sound, studies of injurious behaviour such as tail biting, have to balance the need for information with the welfare of the animals. In the present study definitions of a tail biting outbreak and when to remove a biting pig, had to be decided based on these two factors.

## 5. Conclusions

Providing additional straw in a relatively small amount (7 g/pig/day) on the floor during a tail biting outbreak reduced the risk of an escalation in tail damage more effectively than providing a Bite-Rite, while rope reduced tail damage at an intermediate level which was not significantly different from either straw or Bite-Rite. All three treatments reduced tail directed behaviour on the day of the outbreak compared to the previous day. However, during the seven days recording period after an outbreak, the level of tail directed behaviour increased in pens with Bite-Rite. This increase in tail directed behaviour did not occur in pens with rope and straw. Pigs also interacted more with rope than with Bite-Rite. In general, the results indicate that a Bite-Rite cannot keep pigs interested for very long and it should be combined or rotated with other materials to successfully stop tail biting.

The curative treatments applied in the study were chosen due to their ability to be used under commercial conditions. However, tail damage escalated in approximately one in four straw pens. This indicates that other enrichment treatments or different interventions strategies are needed to more efficiently stop the tail biting behaviour. Future studies comparing the effect of different interventions strategies during a tail biting outbreak are needed to establish this.

## Figures and Tables

**Table 1:** Tail injury classification after Lahrmann et al. [12].

Tail Scoring	Description
Damage severity	
No	No visible tail lesions. The earlier lesion is healed.
Minor scratches	Minor superficial scratches.
Wound	Visible wound and tissue damage larger than a few millimetres in diameter.
Wound—tail end will fall off	The outer part of the tail has almost been bitten off. During healing, the tail tip will fall off.
Damage freshness	
Intact scab	The wound is covered with a hard, dry scab.
Not intact scab	The wound is covered with a scab, but cracks in the scab and dried blood/fresh tissue are visible.
Fresh wound—not bleeding (weeping)	Skin is broken, no scab, no blood—only weeping.
Fresh wound—bleeding	Fresh lesion and fresh blood are visible.
Tail length	
Intact	Full-length tail.
Outer part is missing	The outer part of the tail is missing.
More than half of the tail is missing	More than half of the tail is missing.
<1 cm left of the tail	Less than 1 cm of the tail is left.
Swelling	
No swelling	No swelling.
Swelling is present	Swollen red tail indicating an infection.

**Table 2:** Ethogram describing recorded pigs´ behaviours using scan sampling. Based on by Day et al. [16] and Nannoni et al. [17].

Behaviours	Description
Walking	Standing up on all four legs and showing a clear walking pattern.
Sitting active	The rear end is planted on the floor and the front legs are straight. The pig is actively engaged in one of the behaviours described below.
Lying active	Resting ventrally or laterally on the floor. The pig is actively engaged in one of the behaviours described below.
Object interaction	The head is oriented towards the object and the head is not more than a pig’s length from the object expressing interest. The pig might also manipulate the enrichment with its mouth or snout or perform rooting behaviour.
Wooden stick/chain	Manipulating the wooden sticks or chain with the snout or mouth or using the wooden sticks for other physical purposes like scratching.
Exploring the floor	The snout was touching the floor. The pig performed circling or back and forth head movements or chewing substrate lying on the floor (rooting behaviour).
Other pen objects	Interaction with other pen objects like pen equipment (biting bars, feeder etc.).
Pen mate directed behaviour	Behaviours directed towards pen mates and pigs from the neighbouring pen through the bars. Those behaviours were: snout contact, chasing, headbutting, nosing, snapping at body parts except for the tails, or fighting.

**Table 3:** Ethogram describing tail directed behaviours recorded during continuous sampling. Based on Taylor et al. [18] and Zonderland et al. [19].

Tail Directed Behaviour (TDB)	Description
Tail interest (TI)	The performing pig’s snout was close to and fixated to the recipient’s rear end and making nosing movements.
Tail-in-mouth (TIM)	The performing pig had the recipient’s tail in its mouth or was chewing on it without a response from the recipient.
Two-stage tail biting (TB)	The performing pig had the recipient’s tail in its mouth and was visibly pulling the tail, or the recipient responded to the tail biting with abrupt movements such as (but not exclusively): jumping, turning, running away, changing resting position, and sudden head movement.
Sudden forceful tail biting (TB)	The performing pig was striking out after another pig’s tail suddenly and forcefully. The tail biting was performed without any earlier tail directed sequences such as tail interest. The recipient pig showed similar response as to two-stage tail biting.

**Table 4:** Number of pens, days until outbreak, tail damaged pigs on day 0, number of pens with an escalation in tail damage, and days until escalation presented within treatment.

	Treatment		
	Straw	Rope	Bite-Rite
Pens with curative treatment, *n*	22	20	19
Days till tail biting outbreak ^1^	22 (10.7)	22 (10.0)	20 (10.1)
Tail damaged pigs on day 0, *n* ^1,2^	6.4 (3.1)	6.7 (2.7)	7.2 (4.4)
Pens with an escalation in tail damage, *n*			
four fresh wounds	4	6	11
removed biter	2	1	1
Days till an escalation in tail damage ^1^	21 (11.3)	11 (4.8)	12 (8.9)

^1^ Results are presented as mean (SD). ^2^ Pigs within pen with at least a tail wound. Minor superficial scratches were not encountered as tail damage in the definition of a tail biting outbreak.

**Table 5:** The frequency of behaviours (percentage of pigs) observed on day −1, 0, 2, and 7 and within each treatment (straw, rope and Bite-Rite). Different lowercase letters (a, b and c) indicate significant difference of *p* < 0.05 between days or between enrichment treatment.

Behaviour	Day ^1^						Treatment ^1^				
	−1	0 ^3^	2	7	SE	*p*-Value	Straw	Rope	Bite-Rite	SE	*p*-Value
Active pigs, %	37.0a	39.6b	37.4a	36.8a	0.81	<0.01	38.3	36.7	38.1	1.21	0.49
Object interaction, %	-	23.3a	12.7b	10.2b	1.40	<0.001	-	18.9a	11.9b	1.32	<0.001
Explorative behaviour, %	29.7a	40.5b	36.8c	35.1c	1.03	<0.001	32.7b	39.0a	34.8b	1.34	<0.001
Pen mate directed behaviour except TDB, % ^2^	12.3a	8.26b	9.55c	11.4a	0.56	<0.001	11.1	9.7	10.3	0.84	0.35

^1^ The sum of the recorded behaviours does not sum to 100%, as pigs could be engaged in other behaviours aside from those presented in the table. ^2^ TDB; tail directed behaviour. ^3^ Day 0; the day of the tail biting outbreak.
